# Supplementary material for: Inhibitory interactions promote frequent bistability among competing bacteria
Source: Nat Commun. 2016 Apr 21;7:11274. doi: 10.1038/ncomms11274 (PMC4844671; doi:10.1038/ncomms11274)
Supplement: Supplementary Information — Supplementary Figures 1-8, Supplementary Tables 1-3 and Supplementary References [file ncomms11274-s1.pdf]

Supplementary Figures

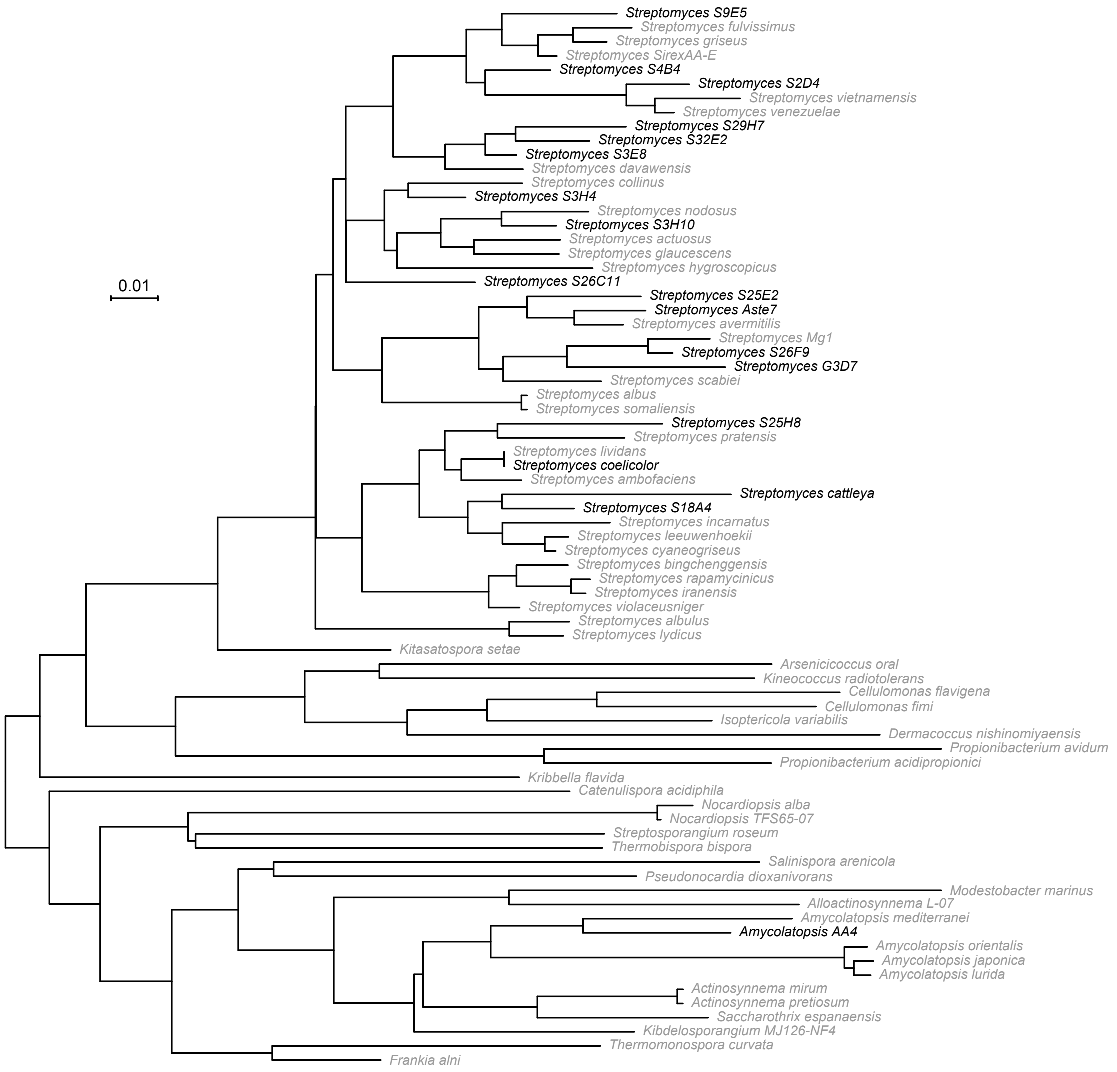

Supplementary Figure 1. Maximum likelihood tree based on 643 nucleotides of the *rpoB* gene belonging to strains used in this study (black labels) and other related strains (gray labels). Strains used in this study cover much of the breadth of known *Streptomyces*. Scale bar shows the expected number of substitutions per site.

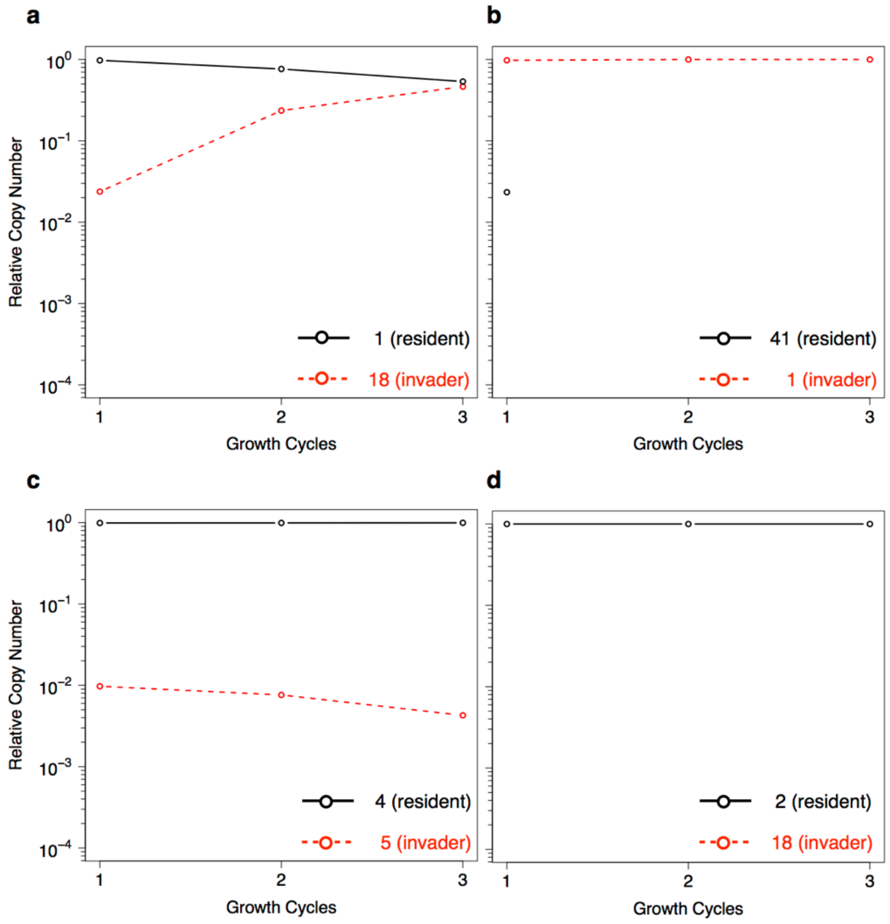

Supplementary Figure 2. Examples of community dynamics during (**a**) a slow invasion, (**b**) a fast invasion, or (**c**, **d**) no invasion. Relative copy number is the fraction of *rpoB* sequencing reads that matched each of the two species at a given growth cycle after background subtraction (see Methods section in the main text). In some cases of non-invasion (**d**) the invader never rose above the lower detection limit, although the presence of the invader could often be visually confirmed in the tube during the first growth cycle (Supplementary Fig. 4). In a subset of 16 of these cases we further confirmed the absence of the invader after the third growth cycle using quantitative PCR, which has a superior lower detection limit (Supplementary Table 3).

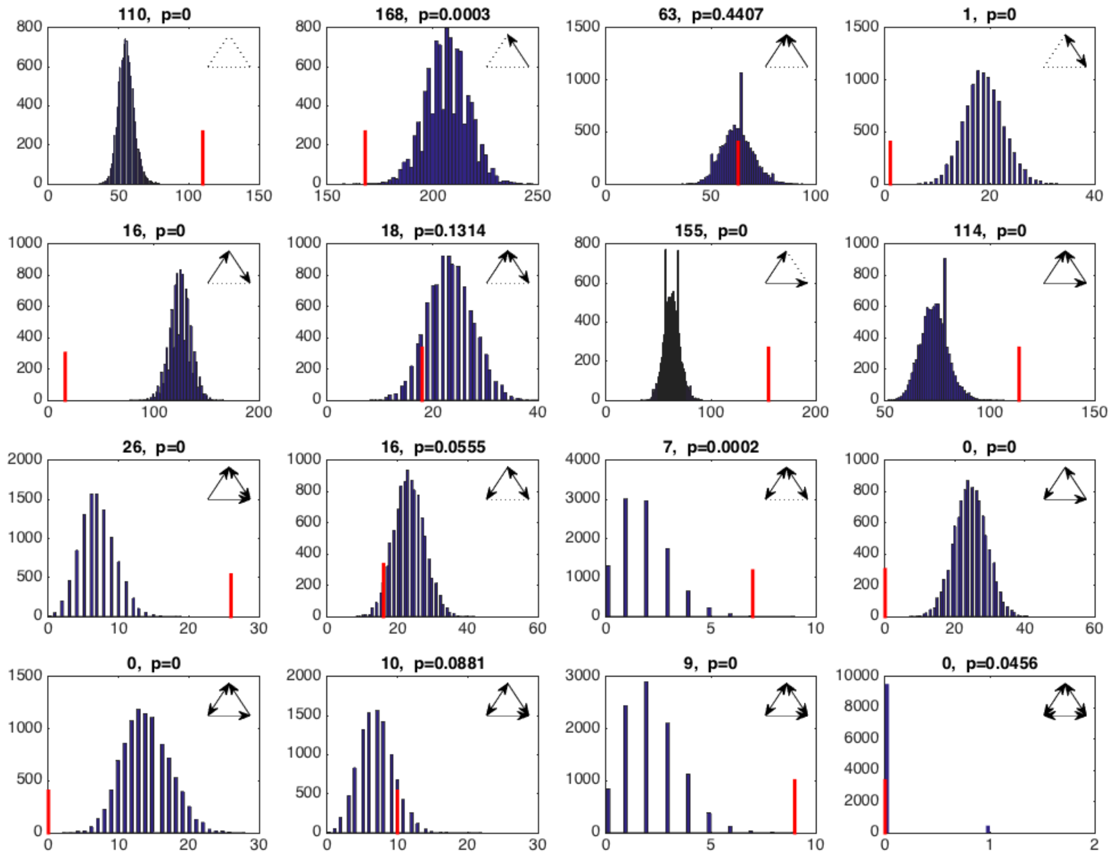

Supplementary Figure 3. Distribution of triplet motifs in randomized invasion networks (blue histograms) relative to the observed number of each motif (red lines). Enrichment for transitivity of hierarchy is evident from histograms (2, 1), (2, 4), (3, 4), and (4, 1) in (row, column) format. Enrichment for transitivity of bistability is evident from histograms (1, 1), (1, 2), and (1, 4). Absence of the 'rock-paper-scissors' dynamic is shown in (3, 4).

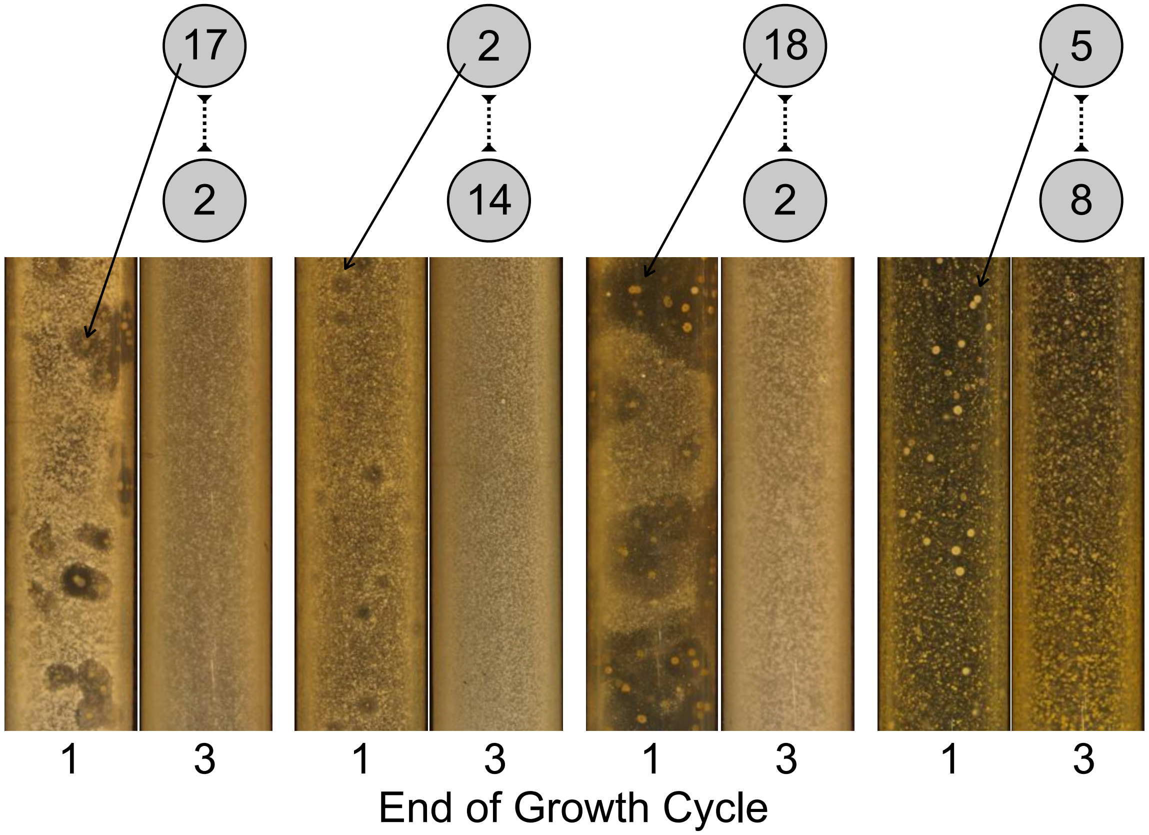

Supplementary Figure 4. Example images of cases where the higher ranked strain in a bistable pairing is visible in the tube after the first growth cycle, but disappears by the third growth cycle.

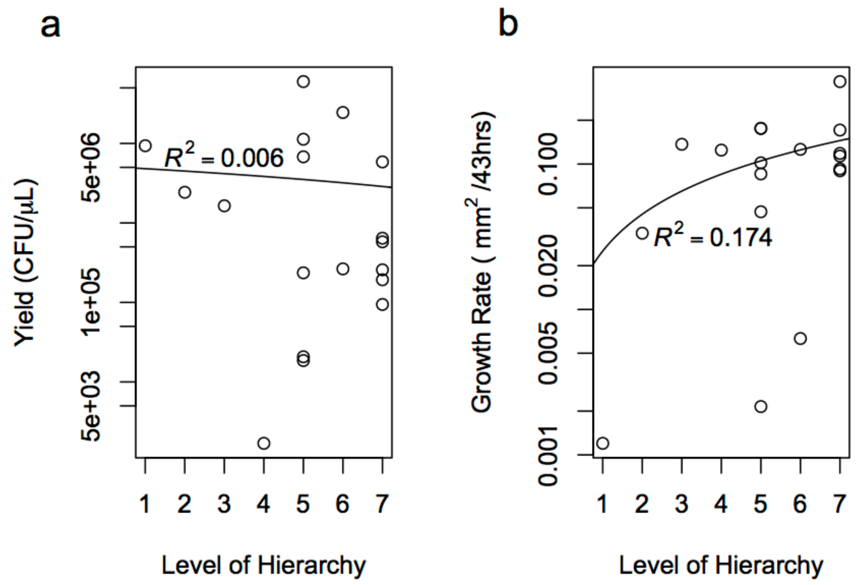

Supplementary Figure 5. Both yield (**a**) and growth rate (**b**) are largely uncorrelated with hierarchy level. Yield was measured for each strain grown by itself after three growth cycles. Colony size was measured under a microscope for separate colonies after 43 hours of growth (see Methods). Note the log-scaled y-axes, which cause the best-fit trend-lines to appear curved.

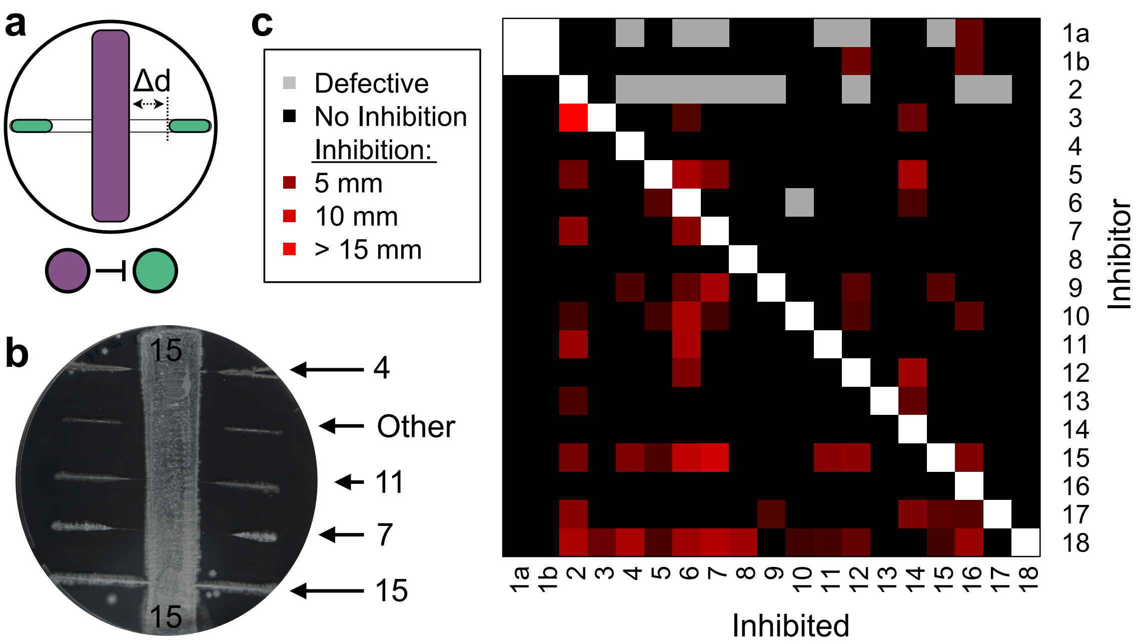

Supplementary Figure 6. Measurement of the inhibition matrix. **a**, Inhibition in the cross-streaking assay was measured as the distance an abundant strain (the inhibitor) was able to prevent sporulation of a less abundant strain intersecting it on a petri dish. **b**, Example experimental results for strain 15 as the inhibitor and four other strains being inhibited. **c**, The matrix of pairwise inhibitions included several strains that were inhibited by most others, and several strains that were not inhibited by any others. The diagonal is white because a strain can grow adjacent to itself (no self-inhibition).

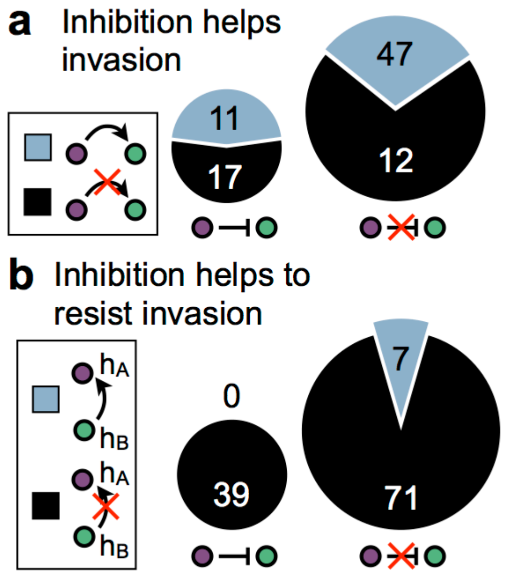

Supplementary Figure 7. Correlations between invasions and inhibitions complementing Fig. 4bc in the main text. **a**, Inhibition appears to increase the likelihood of invasion when using data from all pairs ($p = 0.02$). **b**, Inhibition helps to resist invasion ($p = 0.07$) using a subset in which the resident is at a higher hierarchy level ($3 \geq h_A - h_B > 0$).

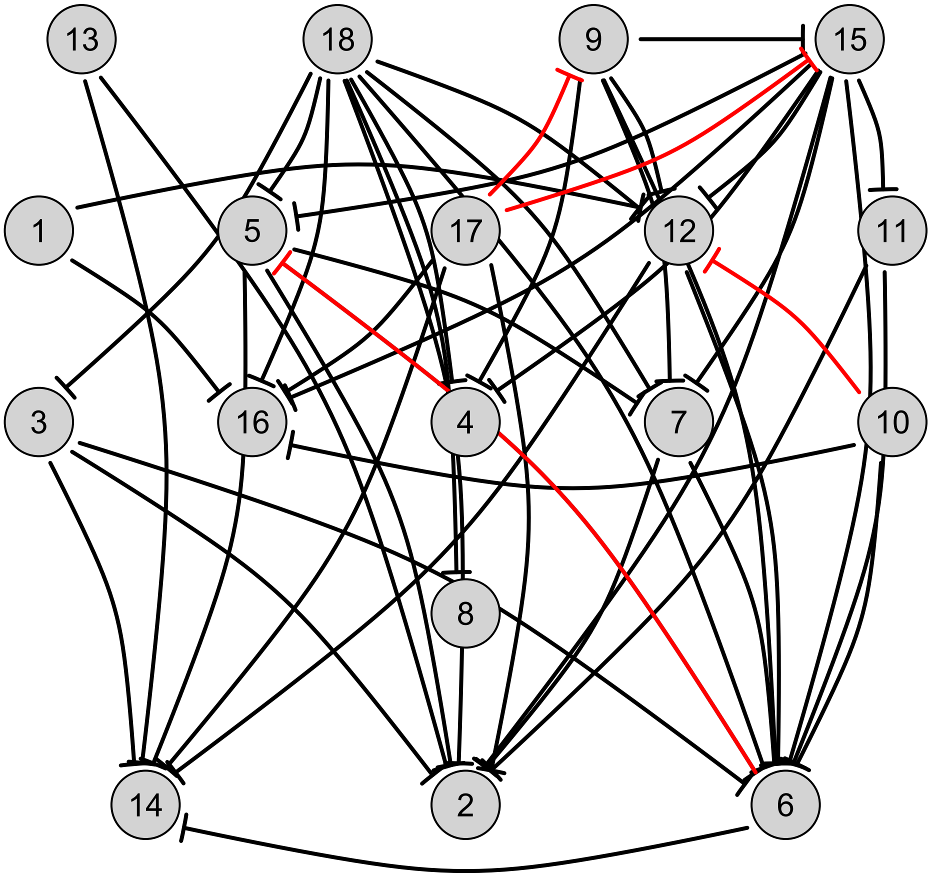

Supplementary Figure 8. One of 11 top scoring alternative hierarchies created by not scoring invasions between pairs that have an inhibitory interaction in either direction. Few inhibitions are directed against the invasion hierarchy, indicating that the downward bias of inhibitions is not entirely due to a role of inhibitions in shaping the invasion hierarchy.

Supplementary Tables

Supplementary Table 1. Strains of *Streptomyces* bacteria used in this study.

Strain Number	Strain Name	Source	Accession	Invader (CFU) $^{\alpha}$	Resident (CFU) $^{\beta}$	Full Rank $^{\gamma}$	Partial Rank $^{\gamma}$
1a	*Amycolatopsis AA4*	[1]	PRJNA33599	132	3.21E+06	6	4.91
1b				139	1.23E+07		
2	*Streptomyces cattleya*	[2]	FQ859185	141	5.64E+06	5	3.18
3	*Streptomyces Aste7*	Owen Park, Madison, WI	KT364455	79	1.18E+05	5	3.91
4	*Strepomyces S25E2*	See Methods	KT364442	131	1.04E+04	5	3.91
5	*Strepomyces S3H4*	See Methods	KT364431	182	2.93E+06	7	4.91
6	*Strepomyces S3H10*	See Methods	KT364432	54	1.21E+06	2	1.45
7	*Strepomyces S26C11*	See Methods	KT364445	14	3.00E+07	5	3.91
8	*Strepomyces S32E2*	See Methods	KT364451	157	8.21E+05	3	2.36
9	*Strepomyces S3E8*	See Methods	KT364429	191	4.71E+04	7	5.91
10	*Strepomyces S29H7*	See Methods	KT364448	235	8.44E+02	4	3.36
11	*Strepomyces S25H8*	See Methods	KT364444	81	1.32E+05	6	4.91
12	*Strepomyces S18A4*	See Methods	KT364439	151	3.39E+06	5	4.91
13	*Streptomyces coelicolor (M145)*	[3]	AL645882	122	1.29E+05	7	5.91
14	*Streptomyces G3D7*	[4]	KT364454	67	4.68E+06	1	1.18
15	*Strepomyces S26F9*	See Methods	KT364446	92	9.64E+04	7	5.91
16	*Strepomyces S9E5*	See Methods	KT364435	297	9.29E+03	5	3.91
17	*Strepomyces S4B4*	See Methods	KT364433	198	3.21E+05	7	4.91
18	*Strepomyces S2D4*	See Methods	KT364427	169	2.89E+05	7	5.91

$^{\alpha}$ Initial concentration as the *invader* strain in Colony Forming Units (CFU) per tube.
$^{\beta}$ Initial concentration as the *resident* strain in Colony Forming Units (CFU) per tube.

[γ] Hierarchical ranking of strains using the full invasion matrix (Full Rank) or only the pairs of strains without inhibition (Partial Rank). The Partial Rank column gives the average of the 11 possible rankings with equivalent scores (see Fig. S7 for one example).

Supplementary Table 2. PCR primers used in this study. For sequencing, the rpoB_amp_F and rpoB_amp_R are the primers used in the first amplification step, and the longer primers are used in the second amplification step. Barcoded primers are named by index (BC) number and, for the reverse primers, a phase offset (+0 to +3 nucleotides)[5].

Primer Name	Primer Sequence (5' to 3')
rpoB_amp_F	AAGGTCGGCCGCTACAAGGT
rpoB_amp_R	GATGTCGTCGGTCTCGAC
rpoB_amp_F1_BC1	CAAGCAGAAGACGGCATACGAGATTAAGAGGCGTGACTGGAGTTCAGACGTGTGCTCTTCCGATCAAGGTCGGCCGCTACAAGGT
rpoB_amp_F1_BC3	CAAGCAGAAGACGGCATACGAGATGCGAATTCGTGACTGGAGTTCAGACGTGTGCTCTTCCGATCAAGGTCGGCCGCTACAAGGT
rpoB_amp_F1_BC4	CAAGCAGAAGACGGCATACGAGATACTGAGCTGTGACTGGAGTTCAGACGTGTGCTCTTCCGATCAAGGTCGGCCGCTACAAGGT
rpoB_amp_F1_BC5	CAAGCAGAAGACGGCATACGAGATTTAGGCACGTGACTGGAGTTCAGACGTGTGCTCTTCCGATCAAGGTCGGCCGCTACAAGGT
rpoB_amp_F1_BC6	CAAGCAGAAGACGGCATACGAGATCTCCGATTGTGACTGGAGTTCAGACGTGTGCTCTTCCGATCAAGGTCGGCCGCTACAAGGT
rpoB_amp_F1_BC7	CAAGCAGAAGACGGCATACGAGATGATGCGAAGTGACTGGAGTTCAGACGTGTGCTCTTCCGATCAAGGTCGGCCGCTACAAGGT
rpoB_amp_F1_BC8	CAAGCAGAAGACGGCATACGAGATCATGGCATGTGACTGGAGTTCAGACGTGTGCTCTTCCGATCAAGGTCGGCCGCTACAAGGT
rpoB_amp_F1_BC9	CAAGCAGAAGACGGCATACGAGATCGTGATCAGTGACTGGAGTTCAGACGTGTGCTCTTCCGATCAAGGTCGGCCGCTACAAGGT
rpoB_amp_F1_BC10	CAAGCAGAAGACGGCATACGAGATTGCATTCCGTGACTGGAGTTCAGACGTGTGCTCTTCCGATCAAGGTCGGCCGCTACAAGGT
rpoB_amp_F1_BC11	CAAGCAGAAGACGGCATACGAGATACGTTCTCGTGACTGGAGTTCAGACGTGTGCTCTTCCGATCAAGGTCGGCCGCTACAAGGT
rpoB_amp_F1_BC12	CAAGCAGAAGACGGCATACGAGATTTCACAGGGTGACTGGAGTTCAGACGTGTGCTCTTCCGATCAAGGTCGGCCGCTACAAGGT
rpoB_amp_F1_BC13	CAAGCAGAAGACGGCATACGAGATAATTGGCCGTGACTGGAGTTCAGACGTGTGCTCTTCCGATCAAGGTCGGCCGCTACAAGGT
rpoB_amp_F1_BC14	CAAGCAGAAGACGGCATACGAGATCCTTACGTGTGACTGGAGTTCAGACGTGTGCTCTTCCGATCAAGGTCGGCCGCTACAAGGT
rpoB_amp_F1_BC15	CAAGCAGAAGACGGCATACGAGATCAGAGGTAGTGACTGGAGTTCAGACGTGTGCTCTTCCGATCAAGGTCGGCCGCTACAAGGT
rpoB_amp_F1_BC16	CAAGCAGAAGACGGCATACGAGATACTCGGTAGTGACTGGAGTTCAGACGTGTGCTCTTCCGATCAAGGTCGGCCGCTACAAGGT
rpoB_amp_F1_BC17	CAAGCAGAAGACGGCATACGAGATGAGTTCCAGTGACTGGAGTTCAGACGTGTGCTCTTCCGATCAAGGTCGGCCGCTACAAGGT
rpoB_amp_F1_BC18	CAAGCAGAAGACGGCATACGAGATTCACGTTCGTGACTGGAGTTCAGACGTGTGCTCTTCCGATCAAGGTCGGCCGCTACAAGGT
rpoB_amp_F1_BC19	CAAGCAGAAGACGGCATACGAGATTATGACCGGTGACTGGAGTTCAGACGTGTGCTCTTCCGATCAAGGTCGGCCGCTACAAGGT
rpoB_amp_F1_BC2	CAAGCAGAAGACGGCATACGAGATATACGCTGGTGACTGGAGTTCAGACGTGTGCTCTTCCGATCAAGGTCGGCCGCTACAAGGT
rpoB_amp_F1_BC20	CAAGCAGAAGACGGCATACGAGATGGCATCATGTGACTGGAGTTCAGACGTGTGCTCTTCCGATCAAGGTCGGCCGCTACAAGGT
rpoB_amp_F1_BC21	CAAGCAGAAGACGGCATACGAGATTCTGGACAGTGACTGGAGTTCAGACGTGTGCTCTTCCGATCAAGGTCGGCCGCTACAAGGT

rpoB_amp_F1_BC22	CAAGCAGAAGACGGCATACGAGATTGAGCAAGGTGACTGGAGTTCAGACGTGTGCTCTTCCGATCAAGGTCGGCCGCTACAAGGT
rpoB_amp_F1_BC23	CAAGCAGAAGACGGCATACGAGATTGACCTGAGTGACTGGAGTTCAGACGTGTGCTCTTCCGATCAAGGTCGGCCGCTACAAGGT
rpoB_amp_F1_BC24	CAAGCAGAAGACGGCATACGAGATCATCTGGAGTGACTGGAGTTCAGACGTGTGCTCTTCCGATCAAGGTCGGCCGCTACAAGGT
rpoB_amp_F1_BC25	CAAGCAGAAGACGGCATACGAGATCGGTCTAAGTGACTGGAGTTCAGACGTGTGCTCTTCCGATCAAGGTCGGCCGCTACAAGGT
rpoB_amp_R1+0_BC1	AATGATACGGCGACCACCGAGATCTACACGCCTCTTAACACTCTTTCCCTACACGACGCTCTTCCGATCTGATGTCGTCGGTCTCGAC
rpoB_amp_R1+1_BC2	AATGATACGGCGACCACCGAGATCTACACCAGCGTATACACTCTTTCCCTACACGACGCTCTTCCGATCTTGATGTCGTCGGTCTCGAC
rpoB_amp_R1+2_BC3	AATGATACGGCGACCACCGAGATCTACACGAATTCGCACACTCTTTCCCTACACGACGCTCTTCCGATCTCTGATGTCGTCGGTCTCGAC
rpoB_amp_R1+3_BC4	AATGATACGGCGACCACCGAGATCTACACAGCTCAGTACACTCTTTCCCTACACGACGCTCTTCCGATCTACAGATGTCGTCGGTCTCGAC
rpoB_amp_R1+0_BC5	AATGATACGGCGACCACCGAGATCTACACGTGCCTAAACACTCTTTCCCTACACGACGCTCTTCCGATCTGATGTCGTCGGTCTCGAC
rpoB_amp_R1+1_BC6	AATGATACGGCGACCACCGAGATCTACACAATCGGAGACACTCTTTCCCTACACGACGCTCTTCCGATCTTGATGTCGTCGGTCTCGAC
rpoB_amp_R1+2_BC7	AATGATACGGCGACCACCGAGATCTACACTTCGCATCACACTCTTTCCCTACACGACGCTCTTCCGATCTCTGATGTCGTCGGTCTCGAC
rpoB_amp_R1+3_BC8	AATGATACGGCGACCACCGAGATCTACACATGCCATGACACTCTTTCCCTACACGACGCTCTTCCGATCTACAGATGTCGTCGGTCTCGAC
rpoB_amp_R1+0_BC9	AATGATACGGCGACCACCGAGATCTACACTGATCACGACACTCTTTCCCTACACGACGCTCTTCCGATCTGATGTCGTCGGTCTCGAC
rpoB_amp_R1+1_BC10	AATGATACGGCGACCACCGAGATCTACACGGAATGCAACACTCTTTCCCTACACGACGCTCTTCCGATCTTGATGTCGTCGGTCTCGAC
rpoB_amp_R1+2_BC11	AATGATACGGCGACCACCGAGATCTACACGAGAACGTACACTCTTTCCCTACACGACGCTCTTCCGATCTCTGATGTCGTCGGTCTCGAC
rpoB_amp_R1+3_BC12	AATGATACGGCGACCACCGAGATCTACACCCTGTGAAACACTCTTTCCCTACACGACGCTCTTCCGATCTACAGATGTCGTCGGTCTCGAC
rpoB_amp_R1+0_BC13	AATGATACGGCGACCACCGAGATCTACACGGCCAATTACACTCTTTCCCTACACGACGCTCTTCCGATCTGATGTCGTCGGTCTCGAC
rpoB_amp_R1+1_BC14	AATGATACGGCGACCACCGAGATCTACACACGTAAGGACACTCTTTCCCTACACGACGCTCTTCCGATCTTGATGTCGTCGGTCTCGAC
rpoB_amp_R1+2_BC15	AATGATACGGCGACCACCGAGATCTACACTACCTCTGACACTCTTTCCCTACACGACGCTCTTCCGATCTCTGATGTCGTCGGTCTCGAC
rpoB_amp_R1+3_BC16	AATGATACGGCGACCACCGAGATCTACACTACCGAGTACACTCTTTCCCTACACGACGCTCTTCCGATCTACAGATGTCGTCGGTCTCGAC
rpoB_amp_R1+0_BC17	AATGATACGGCGACCACCGAGATCTACACTGGAACTCACACTCTTTCCCTACACGACGCTCTTCCGATCTGATGTCGTCGGTCTCGAC
rpoB_amp_R1+1_BC18	AATGATACGGCGACCACCGAGATCTACACGAACGTGAACACTCTTTCCCTACACGACGCTCTTCCGATCTTGATGTCGTCGGTCTCGAC
rpoB_amp_R1+2_BC19	AATGATACGGCGACCACCGAGATCTACACCGGTCATAACACTCTTTCCCTACACGACGCTCTTCCGATCTCTGATGTCGTCGGTCTCGAC
rpoB_amp_R1+3_BC20	AATGATACGGCGACCACCGAGATCTACACATGATGCCACACTCTTTCCCTACACGACGCTCTTCCGATCTACAGATGTCGTCGGTCTCGAC
rpoB_amp_R1+0_BC21	AATGATACGGCGACCACCGAGATCTACACTGTCCAGAACACTCTTTCCCTACACGACGCTCTTCCGATCTGATGTCGTCGGTCTCGAC
rpoB_amp_R1+1_BC22	AATGATACGGCGACCACCGAGATCTACACCTTGCTCAACACTCTTTCCCTACACGACGCTCTTCCGATCTTGATGTCGTCGGTCTCGAC
rpoB_amp_R1+2_BC23	AATGATACGGCGACCACCGAGATCTACACTCAGGTCAACACTCTTTCCCTACACGACGCTCTTCCGATCTCTGATGTCGTCGGTCTCGAC

rpoB_amp_R1+3_BC24	AATGATACGGCGACCACCGAGATCTACACTCCAGATGACACTCTTTCCCTACACGACGCTCTTCCGATCTACAGATGTCGTCGGTCTCGAC
rpoB_amp_R1+0_BC25	AATGATACGGCGACCACCGAGATCTACACTTAGACCGACACTCTTTCCCTACACGACGCTCTTCCGATCTGATGTCGTCGGTCTCGAC
rpoB_strain2_F1	GGCCGGTCTGGACGTG
rpoB_strain2_R1	GCGGTCAGGTAGTGCACGT
rpoB_strain9_F1	GGTCAGGTCACCGACGAC
rpoB_strain9_R1	GTGAAGCGCATGTCGTCATTG
rpoB_strain11_F1	GGGCCGCGAGATCATCG
rpoB_strain11_R1	CGACGCCACCACGGG
rpoB_strain15_F1	TCATCGACGGCGTCGTCA
rpoB_strain15_R1	CATGTCCTCGGACAGGGC
rpoB_strain17_F1	CCGTCTCTCGGCGCTC
rpoB_strain17_R1	GCTCGTCGTTCAGGGTCG
rpoB_strain18_F1	CGGCGAGAACGGCAACGAG
rpoB_strain18_R1	CCGGATGTTGATCAGGGTCTGA

Supplementary Table 3. Results of quantitative PCR amplification of a subset of 8 bistable pairs after the third growth cycle.

Bistable pairs		Threshold cycle (C_t)					
Resident	Invader	Resident C_t	Invader C_t	ΔC_t	Melt curve $^{\alpha}$	Gel run $^{\beta}$	Sanger Sequencing $^{\gamma}$
9	17	17.48	36.49	19.01	Different	Matched	Artifact
17	9	15.14	29.48	14.34	Different	Different	N/A
15	18	15.90	34.59	18.69	Different	Different	N/A
18	15	14.09	34.92	20.83	Different	Different	N/A
15	9	17.03	31.35	14.32	Different	Absent	N/A
9	15	17.01	37.23	20.22	Different	Matched	Artifact
2	17	18.65	39.38	20.73	Different	Different	N/A
17	2	16.30	22.04	5.74	Different	Different	N/A
2	11	24.08	40.14	16.06	Different	Different	N/A
11	2	17.93	26.75	8.82	Different	Different	N/A
15	17	16.09	44.29	28.20	Different	Absent	N/A
17	15	17.72	36.88	19.16	Different	Absent	N/A
17	18	16.73	34.15	17.42	Different	Absent	N/A
18	17	14.57	36.63	22.06	Different	Absent	N/A
2	18	16.31	37.60	21.29	Different	Absent	N/A
18	2	15.21	26.97	11.76	Different	Absent	N/A

$^{\alpha}$ Whether the shape of the melt curve matched or was different than would be expected if the invader's target DNA had amplified.

$^{\beta}$ Whether the amplicon length matched or was different than would be expected if the invader's target DNA had amplified. "Absent" indicates that the gel run band was too short to appear on the gel (< ~70 base pairs).

$^{\gamma}$ Whether the results of Sanger sequencing matched the invader's target DNA, appeared to be a PCR artifact (Artifact), or was not sequenced (N/A).

Supplementary References

1. Seyedsayamdost, M. R., Traxler, M. F., Zheng, S.-L., Kolter, R. & Clardy, J. Structure and Biosynthesis of Amychelin, an Unusual Mixed-Ligand Siderophore from Amycolatopsis sp.AA4. *J. Am. Chem. Soc.* **133,** 11434–11437 (2011).
2. Barbe, V. *et al.* Complete Genome Sequence of Streptomyces cattleya NRRL 8057, a Producer of Antibiotics and Fluorometabolites. *Journal of Bacteriology* **193,** 5055–5056 (2011).
3. Bentley, S. D. *et al.* Complete genome sequence of the model actinomycete Streptomyces coelicolor A3 (2). *Nature* **417,** 141–147 (2002).
4. Vetsigian, K., Jajoo, R. & Kishony, R. Structure and Evolution of Streptomyces Interaction Networks in Soil and In Silico. *Plos Biol* **9,** e1001184 (2011).
5. Wu, L. *et al.* Phasing amplicon sequencing on Illumina Miseq for robust environmental microbial community analysis. *BMC Microbiology* 1–12 (2015). doi:10.1186/s12866-015-0450-4
